# Understanding Alternative Bullying Perspectives Through Research Engagement With Young People

**DOI:** 10.3389/fpsyg.2019.01984

**Published:** 2019-08-28

**Authors:** Niamh O’Brien

**Affiliations:** School of Education and Social Care, Faculty of Health, Education, Medicine and Social Care, Anglia Ruskin University, Chelmsford, United Kingdom

**Keywords:** bullying, young people, participatory research, social constructionism, young people as researchers, collaboration, bullying supports

## Abstract

Bullying research has traditionally been dominated by largescale cohort studies focusing on the personality traits of bullies and victims. These studies focus on bullying prevalence, risk and protective factors, and negative outcomes. A limitation of this approach is that it does not explain why bullying happens. Qualitative research can help shed light on these factors. This paper discusses the findings from four mainly qualitative research projects including a systematic review and three empirical studies involving young people to various degrees within the research process as respondents, co-researchers and commissioners of research. Much quantitative research suggests that young people are a homogenous group and through the use of surveys and other large scale methods, generalizations can be drawn about how bullying is understood and how it can be dealt with. Findings from the studies presented in this paper, add to our understanding that young people appear particularly concerned about the role of wider contextual and relational factors in deciding if bullying has happened. These studies underscore the relational aspects of definitions of bullying and, how the dynamics of young people’s friendships can shift what is understood as bullying or not. Moreover, to appreciate the relational and social contexts underpinning bullying behaviors, adults and young people need to work together on bullying agendas and engage with multiple definitions, effects and forms of support. Qualitative methodologies, in particular participatory research opens up the complexities of young lives and enables these insights to come to the fore. Through this approach, effective supports can be designed based on what young people want and need rather than those interpreted as supportive through adult understanding.

## Introduction

Research on school bullying has developed rapidly since the 1970s. Originating in social and psychological research in Norway, Sweden, and Finland, this body of research largely focusses on individualized personality traits of perpetrators and victims (Olweus, 1995). Global interest in this phenomenon subsequently spread and bullying research began in the United Kingdom, Australia, and the United States (Griffin and Gross, 2004). Usually quantitative in nature, many studies examine bullying prevalence, risk and protective factors, and negative outcomes (Patton et al., 2017). Whilst quantitative research collates key demographic information to show variations in bullying behaviors and tendencies, this dominant bullying literature fails to explain *why* bullying happens. Nor does it attempt to understand the wider social contexts in which bullying occurs. Qualitative research on the other hand, in particular participatory research, can help shed light on these factors by highlighting the complexities of the contextual and relational aspects of bullying and the particular challenges associated with addressing it. Patton et al. (2017) in their systematic review of qualitative methods used in bullying research, found that the use of such methods can enhance academic and practitioner understanding of bullying.

In this paper, I draw on four bullying studies; one systematic review of both quantitative and qualitative research (O’Brien, 2009) and three empirical qualitative studies (O’Brien and Moules, 2010; O’Brien, 2016, 2017) (see Table 1 below). I discuss how participatory research methodologies, to varying degrees, were used to facilitate bullying knowledge production among teams of young people and adults. Young people in these presented studies were consequently involved in the research process along a continuum of involvement (Bragg and Fielding, 2005). To the far left of the continuum, young people involved in research are referred to as “active respondents” and their data informs teacher practice. To the middle of the continuum sit “students as co-researchers” who work with teachers to explore an issue which has been identified by that teacher. Finally to the right, sit “students as researchers” who conduct their own research with support from teachers. Moving from left to right of the continuum shows a shift in power dynamics between young people and adults where a partnership develops. Young people are therefore recognized as equal to adults in terms of what they can bring to the project from their own unique perspective, that of being a young person now.

**TABLE 1:** The studies.

**The study**	**Title**	**Author(s) and year**	**Design**	**Methods**	**Participants**	**Position of study on Bragg and Fielding, 2005 continuum**	**Analytical framework**	**Publication from study**
Study 1	Secondary school teachers’ and pupils’ definitions of bullying in the United Kingdom: a systematic review	O’Brien, 2009	Systematic Review	Systematic literature review of five papers: • Two quantitative studies • One mixed methods study • One qualitative study • One quantitative study with a qualitative aspect	3,283 pupils, 225 teachers	Study sits to the far left of the continuum, as young people were not directly involved as “active respondents” but their views were heard through secondary data analysis.	Thematic Analysis, Braun and Clarke, 2006. In the case of the extracted quantitative data, Popay et al., 2006 claim that the variables incorporated in surveys can be extracted as “themes” similar to conceptual themes extracted from qualitative research.	O’Brien, 2009. Secondary school teachers’ and pupils’ definitions of bullying in the United Kingdom: a systematic review. *Evidence and Policy*, 5(4), pp. 399–426.
Study 2	The impact of cyber-bullying on young people’s mental health	O’Brien and Moules, 2010	Participatory Research	Online questionnaire (open questions), focus groups	490 young people and responses from 11 schools	Study shifts between the middle of the continuum: “students as co-researchers” and right: “students as researchers”	Thematic Analysis, Braun and Clarke, 2006	O’Brien and Moules, 2013. Not sticks and stones but tweets and texts: findings from a national cyberbullying project. *Pastoral Care in Education*, 31(1), pp. 53-65.
Study 3	To “Snitch” or Not to “Snitch”? Using PAR to Explore Bullying in a Private Day and Boarding School.	O’Brien, 2016	Participatory Action Research (PAR)	Online questionnaire (open questions), focus groups, student led interviews, paper questionnaires	155 students, 135 parents, 12 school staff members	Study shifts between the middle of the continuum: “students as co-researchers” and right: “students as researchers”	Thematic Analysis, Braun and Clarke, 2006	O’Brien, 2014. “I didn’t want to be known as a snitch”: Using PAR to explore bullying in a private day and boarding school. *Childhood Remixed.* Conference Edition, February, 2014, University Campus Suffolk. pp. 86–96. O’Brien et al., 2018a. Negotiating the research space between young people and adults in a PAR study exploring school bullying. In M. Torronen., C. Munn-Giddings, C., and L. Tarkiainen (eds), Reciprocal Relationships and Well-Being: Implications for Social Work and Social Policy. Oxon: Routledge. Pp. 160-175. O’Brien et al., 2018b. The repercussions of reporting bullying: some experiences of students at an independent secondary school. *Pastoral Care in Education*, 36(1), pp. 29–43. O’Brien et al., 2018c. The ethics of involving young people directly in the research process. *Childhood Remixed.* Conference Edition, May 2018, pp. 115–128. ISSN 2515–4516 (online) Journal homepage www.uos.ac.uk/content/center-for-study-children-childhood
Study 4	An exploratory study of bullied young people’s self-exclusion from school	O’Brien, 2017	Qualitative research	Interviews	4 young people, 2 parents	Study sits to the left of the continuum, as young people were involved as “active respondents” in informing adult understanding of the issue.	Thematic Analysis, Braun and Clarke, 2006	O’Brien, 2017. An exploratory study of bullied young people’s self-exclusion from school *Evidence: presented at meetings of the All Party Parliamentary Group on Bullying 2011–2016.* Available from: http://arro.anglia.ac.uk/id/eprint/702024

In this paper, I advocate for the active involvement of young people in the research process in order to enhance bullying knowledge. Traditional quantitative studies have a tendency to homogenize young people by suggesting similarity in thinking about what constitutes bullying. However, qualitative studies have demonstrated that regardless of variables, young people understand bullying in different ways so there is a need for further research that starts from these perspectives and focusses on issues that young people deem important. Consequently, participatory research allows for the stories of the collective to emerge without losing the stories of the individual, a task not enabled through quantitative approaches.

## What Is Bullying?

Researching school bullying has been problematic and is partly related to the difficulty in defining it (Espelage, 2018). Broadly speaking, bullying is recognized as aggressive, repeated, intentional behavior involving an imbalance of power aimed toward an individual or group of individuals who cannot easily defend themselves (Vaillancourt et al., 2008). In more recent times, “traditional” bullying behaviors have been extended to include cyber-bullying, involving the use of the internet and mobile-phones (Espelage, 2018). Disagreements have been noted in the literature about how bullying is defined by researchers linked to subject discipline and culture. Some researchers for example, disagree about the inclusion or not of repetition in definitions (Griffin and Gross, 2004) and these disagreements have had an impact on interpreting findings and prevalence rates. However, evidence further suggests that young people also view bullying in different ways (Guerin and Hennessy, 2002; Cuadrado-Gordillo, 2012; Eriksen, 2018). Vaillancourt et al. (2008) explored differences between researchers and young people’s definitions of bullying, and found that children’s definitions were usually spontaneous, and did not always encompass the elements of repetition, power imbalance and intent. They concluded, that children need to be provided with a bullying definition so similarities and comparisons can be drawn. In contrast, Huang and Cornell (2015) found no evidence that the inclusion of a definition effected prevalence rates. Their findings, they suggest, indicate that young people use their own perceptions of bullying when answering self-report questionnaires and they are not influenced by an imposed definition.

Nevertheless, differences in children and young people’s bullying definitions are evident in the research literature and have been explained by recourse to age and stage of development (Smith et al., 2002) and their assumed lack of understanding about what constitutes bullying (Boulton and Flemington, 1996). Naylor et al. (2001) for example, found that younger children think similarly in their definitions of bullying, while Smith et al. (2002) found that 8 year olds did not distinguish as clearly between different forms of behavioral aggression as 14 year olds. Methodological limitations associated with understanding bullying have been identified by Forsberg et al. (2018) and Maunder and Crafter (2018). These authors postulate that quantitative approaches, although providing crucial insights in understanding bullying, are reliant on pre-defined variables, which can shield some of the complexities that qualitative designs can unravel, as individual experiences of bullying are brought to the fore. Indeed, La Fontaine (1991) suggests that unlike standard self-report questionnaires and other quantitative methods used to collect bullying data, analyzing qualitative data such as those collected from a helpline, enables the voice of young people to be heard and consequently empowers adults to understand bullying on their terms rather than relying solely on interpretations and perceptions of adults. Moore and Maclean (2012) collected survey, as well as interview and focus group data, on victimization occurring on the journey to and from school. They found that what young people determined as victimization varied and was influenced by a multifaceted array of circumstances, some of which adults were unaware of. Context for example, played an important role where certain behaviors in one situation could be regarded as victimization while in another they were not. Specific behaviors including ignoring an individual was particularly hurtful and supporting a friend who was the subject of victimization could lead to their own victimization.

Lee (2006) suggests that some bullying research does not reflect individual experiences, and are thus difficult for participants to relate to. Canty et al. (2016) reiterates this and suggests that when researchers provide young people with bullying definitions in which to position their own experiences, this can mask some of the complexities that the research intends to uncover. Such approaches result in an oversight into the socially constructed and individual experiences of bullying (Eriksen, 2018). Griffin and Gross (2004) further argue that when researchers use vague or ambiguous definitions an “overclassification of children as bullies or victims” (p. 381) ensues. Consequently, quantitative research does not consider children as reliable in interpreting their own lived experiences and therefore some of the interactions they consider as bullying, that do not fit within the conventional definitions, are concealed. This approach favors the adult definition of bullying regarding it as “more reliable” than the definitions of children and young people Canty et al. (2016). The perceived “seriousness” of bullying has also been explored. Overall, young people and adults are more likely to consider direct bullying (face-to-face actions including hitting, threatening and calling names) as “more serious” than indirect bullying (rumor spreading, social exclusion, forcing others to do something they do not want to do) (Maunder et al., 2010; Skrzypiec et al., 2011). This perception of “seriousness,” alongside ambiguous definitions of bullying, has further implications for reporting it. Despite the advice given to young people to report incidents of school bullying (Moore and Maclean, 2012), the literature suggests that many are reluctant to do so (deLara, 2012; Moore and Maclean, 2012).

Several factors have been highlighted as to why young people are reluctant to report bullying (Black et al., 2010). deLara (2012), found apprehension in reporting bullying to teachers due to the fear that they will either not do enough or too much and inadvertently make the situation worse, or fear that teachers will not believe young people. Research also shows that young people are reluctant to tell their parents about bullying due to perceived over-reaction and fear that the bullying will be reported to their school (deLara, 2012; Moore and Maclean, 2012). Oliver and Candappa (2007) suggest that young people are more likely to confide in their friends than adults (see also Moore and Maclean, 2012; Allen, 2014). However, if young people believe they are being bullied, but are unable to recognize their experiences within a predefined definition of bullying, this is likely to impact on their ability to report it.

Research from psychology, sociology, education and other disciplines, utilizing both quantitative and qualitative approaches, have enabled the generation of bullying knowledge to date. However, in order to understand *why* bullying happens and how it is influenced by wider social constructs there is a need for further qualitative studies, which hear directly from children and young people themselves. The next section of this paper discusses the theoretical underpinnings of this paper, which recognizes that young people are active agents in generating new bullying knowledge alongside adults.

## Theoretical Underpinnings – Hearing From Children and Young People

The sociology of childhood (James, 2007; Tisdall and Punch, 2012) and children’s rights agenda more broadly (United Nations Convention on the Rights of the Child, 1989) have offered new understandings and methods for research which recognize children and young people as active agents and experts on their own lives. From this perspective, research is conducted *with* rather than *on* children and young people (Kellett, 2010).

Participatory methodologies have proven particularly useful for involving young people in research as co-researchers (see for example O’Brien and Moules, 2007; Stoudt, 2009; Kellett, 2010; Spears et al., 2016). This process of enquiry actively involves those normally being studied in research activities. Previously, “traditional” researchers devalued the experiences of research participants arguing that due to their distance from them, they themselves are better equipped to interpret these experiences (Beresford, 2006). However, Beresford (2006) suggests that the shorter the distance between direct experience and interpretation, the less distorted and inaccurate the resulting knowledge is likely to be. Jones (2004) further advocates that when young people’s voices are absent from research about them the research is incomplete. Certainly Spears et al. (2016), adopted this approach in their study with the Young and Well Cooperative Research Centre (CRC) in Australia. Young people played an active role within a multidisciplinary team alongside researchers, practitioners and policymakers to co-create and co-evaluate the learning from four marketing campaigns for youth wellbeing through participatory research. Through this methodological approach, findings show that young people were able to reconceptualize mental health and wellbeing from their own perspectives as well as share their lived experiences with others (Spears et al., 2016). Bland and Atweh (2007), Ozer and Wright (2012), highlight the benefits afforded to young people through this process, including participating in dialog with decision-makers and bringing aspects of teaching and learning to their attention.

Against this background, data presented for this paper represents findings from four studies underpinned by the ethos that bullying is socially constructed and is best understood by exploring the context to which it occurs (Schott and Sondergaard, 2014; Eriksen, 2018). This socially constructed view focusses on the evolving positions within young people’s groups, and argues that within a bullying situation sometimes a young person is the bully, sometimes the victim and sometimes the bystander/witness, which contrasts the traditional view of bullying (Schott and Sondergaard, 2014). The focus therefore is on group relationships and dynamics. For that reason, Horton (2011) proposes that if bullying is an extensive problem including many young people, then focusing entirely on personality traits will not generate new bullying knowledge and will be problematic in terms of interventions. It is important to acknowledge that this change in focus and view of bullying and how it is manifested in groups, does not negate the individual experiences of bullying rather the focus shifts to the process of being accepted, or not, by the group (Schott and Sondergaard, 2014).

## The Studies

This section provides a broad overview of the four included studies underpinned by participatory methodologies. Table 1 presents the details of each study. Young people were involved in the research process as respondents, co-researchers and commissioners of research, along a continuum as identified by Bragg and Fielding (2005). This ranged from “active respondents” to the left of the continuum, “students as co-researchers” in the middle and “students as researchers” to the right of the continuum. Young people were therefore recognized as equal to adults in terms of what they can bring to the project from their own unique perspectives (Bradbury-Jones et al., 2018).

A key finding from study one (O’Brien, 2009) was the *lack* of voice afforded to young people through the research process and can be seen to reflect the far left of Bragg and Fielding (2005) continuum, as young people were not directly involved as “active respondents” but their views were included in secondary data analysis and informed the studies that followed. For example, the quantitative studies used an agreed academic definition of bullying which may or may not have influenced how young participants defined bullying within the studies. On the other hand, the qualitative study involved a group of students in deciding which questions to ask of the research participants and in interpreting the findings.

In contrast, study two (O’Brien and Moules, 2010) was commissioned and led by a group of young people called PEAR (Public health, Education, Awareness, Researchers), who were established to advise on public health research in England. PEAR members were based in two large English cities and comprised 20 young people aged between 13 and 20 years. The premise of the study was that PEAR members wanted to commission research into cyber bullying and the effects this has on mental health from the perspectives of young people rather than adult perspectives. This project was innovative as young people commissioned the research and participated as researchers (Davey, 2011) and can be seen to reflect the middle “students as co-researchers” as well as moving toward to right “students as researchers” of Bragg and Fielding (2005) continuum. Although the young people did not carry out the day-to-day work on the project, they were responsible for leading and shaping it. More importantly, the research topic and focus were decided with young people and adults together.

Study three (O’Brien, 2016) involved five self-selecting students from an independent day and boarding school who worked with me to answer this question: *What do young people in this independent day and boarding school view as the core issue of bullying in the school and how do they want to address this?* These students called themselves R4U (Research for You) with the slogan *researching for life without fear*. Three cycles of Participatory Action Research (PAR) ensued, where decision making about direction of the research, including methods, analysis and dissemination of findings were made by the research team. As current students of the school, R4U had a unique “insider knowledge” that complemented my position as the “academic researcher.” By working together to generate understanding about bullying at the school, the findings thus reflected this diversity in knowledge. As the project evolved so too did the involvement of the young researchers and my knowledge as the “outsider” (see O’Brien et al., 2018a for further details). Similar to study two, this project is situated between the middle: “students as co-researchers” and the right: “students as researchers” of Bragg and Fielding (2005) continuum.

Study four (O’Brien, 2017) was small-scale and involved interviewing four young people who were receiving support from a charity providing therapeutic and educational support to young people who self-exclude from school due to anxiety, as a result of bullying. Self-exclusion, for the purposes of this study, means that a young person has made a decision not to go to school. It is different from “being excluded” or “truanting” because these young people do not feel safe at school and are therefore too anxious to attend. Little is known about the experiences of young people who self-exclude due to bullying and this study helped to unravel some of these issues. This study reflects the left of Bragg and Fielding (2005) continuum where the young people were involved as “active respondents” in informing adult understanding of the issue.

A variety of research methods were used across the four studies including questionnaires, interviews and focus groups (see Table 1 for more details). In studies two and three, young researchers were fundamental in deciding the types of questions to be asked, where they were asked and who we asked. In study three the young researchers conducted their own peer-led interviews. The diversity of methods used across the studies are a strength for this paper. An over-reliance on one method is not portrayed and the methods used reflected the requirements of the individual studies.

## Informed Consent

Voluntary positive agreement to participate in research is referred to as “consent” while “assent,” refers to a person’s compliance to participate (Coyne, 2010). The difference in these terms are normally used to distinguish the “legal competency of children over and under 16 years in relation to research.” (Coyne, 2010, 228). In England, children have a legal right to consent so therefore assent is non-applicable (Coyne, 2010). However, there are still tensions surrounding the ability of children and young people under the age of 18 years to consent in research which are related to their vulnerability, age and stage of development (Lambert and Glacken, 2011). The research in the three empirical studies (two, three and four) started from the premise that all young participants were competent to consent to participate and took the approach of Coyne (2010) who argues that parental/carer consent is not always necessary in social research. University Research Ethics Committees (RECs) are nonetheless usually unfamiliar with the theoretical underpinnings that children are viewed as social actors and generally able to consent for themselves (Lambert and Glacken, 2011; Fox, 2013; Parsons et al., 2015).

In order to ensure the young people in these reported studies were fully informed of the intentions of each project and to adhere to ethical principles, age appropriate participant information sheets were provided to all participants detailing each study’s requirements. Young people were then asked to provide their own consent by signing a consent form, any questions they had about the studies were discussed. Information sheets were made available to parents in studies three and four. In study two, the parents of young people participating in the focus groups were informed of the study through the organizations used to recruit the young people. My full contact details were provided on these sheets so parents/carers could address any queries they had about the project if they wished. When young people participated in the online questionnaire (study two) we did not know who they were so could not provide separate information to parents. Consequently, all participants were given the opportunity to participate in the research without the consent of their parents/carers unless they were deemed incompetent to consent. In this case the onus was on the adult (parent or carer for example) to prove incompetency (Alderson, 2007). Favorable ethical approval, including approval for the above consent procedures, was granted by the Faculty Research Ethics Committee at Anglia Ruskin University.

In the next section I provide a synthesis of the findings across the four studies before discussing how participatory research with young people can offer new understandings of bullying and its impacts on young people.

## Findings

Although each study was designed to answer specific bullying research questions, the following key themes cut across all four studies^1^ :

• Bullying definitions
  - Behaviors
• Impact of bullying on victim
• Reporting bullying

### Bullying Definitions

#### Behaviors

Young people had various understandings about what they considered bullying to be. Overall, participants agreed that aggressive direct behaviors, mainly focusing on physical aggression, constituted bullying:

“…if someone is physically hurt then that is bullying straight away.” (Female, study 3).

“I think [cyber-bullying is] not as bad because with verbal or physical, you are more likely to come in contact with your attacker regularly, and that can be disturbing. However, with cyber-bullying it is virtual so you can find ways to avoid the person.” (Female, study 2).

Name-calling was an ambiguous concept, young people generally believed that in isolation name-calling might not be bullying behavior or it could be interpreted as “joking” or “banter”:

“I never really see any, a bit of name calling and taking the mick but nothing ever serious.” (Male, study 3).

The concept of “banter” or “joking” was explored in study three as a result of the participatory design. Young people suggested “banter” involves:

“…a personal joke or group banter has no intention to harm another, it is merely playful jokes.” (Female, study 3).

However, underpinning this understanding of “banter” was the importance of intentionality:

“Banter saying things bad as a joke and everyone knows it is a joke.” (Male, study 3).

“Banter” was thus contentious when perception and reception were ambiguous. In some cases, “banter” was considered “normal behavior”:

“…we’ve just been joking about, but it’s never been anything harsh it’s just been like having a joke…” (Male, study 3).

The same view was evident in relation to cyber-bullying. Some participants were rather dismissive of this approach suggesting that it did not exist:

“I don’t really think it exists. If you’re being cyber-“bullied” then there is something wrong with you- it is insanely easy to avoid, by blocking people and so on. Perhaps it consists of people insulting you online?” (Male, study 2).

When young people considered additional factors added to name calling such as the type of name-calling, or aspects of repetition or intention, then a different view was apparent.

“…but it has to be constant it can’t be a single time because that always happens.” (Male, study 3).

Likewise with words used on social media, young people considered intentionality in their consideration of whether particular behaviors were bullying, highlighting important nuances in how bullying is conceptualized:

“Some people they don’t want to sound cruel but because maybe if you don’t put a smiley face on it, it might seem cruel when sometimes you don’t mean it.” (Female, study 2).

Study one also found that young people were more likely to discuss sexist or racist bullying in interviews or focus groups but this information was scarce in the questionnaire data. This is possibly as a result of how the questions were framed and the researchers’ perspectives informing the questions.

Evident across the four studies was the understanding young people had about the effects of continuous name-calling on victims:

“…you can take one comment, you can just like almost brush it off, but if you keep on being bullied and bullied and bullied then you might kind of think, hang on a minute, they’ve taken it a step too far, like it’s actually become more personal, whereas just like a cheeky comment between friends it’s become something that’s more serious and more personal and more annoying or hurtful to someone.” (Female, study 3).

“Cyber-bullying is basically still verbal bullying and is definitely psychological bullying. Any bullying is psychological though, really. And any bullying is going to be harmful.” (Female, study 2).

Aspects of indirect bullying (social exclusion) were features of studies one and three. For the most part, the research reviewed in study one found that as young people got older they were less likely to consider characteristics of social exclusion in their definitions of bullying. In study three, when discussing the school’s anti-bullying policy, study participants raised questions about “*isolating a student from a friendship group*.” Some contested this statement as a form of bullying:

“…. there is avoiding, as in, not actively playing a role in trying to be friends which I don’t really see as bullying I see this as just not getting someone to join your friendship group. Whereas if you were actually leaving him out and rejecting him if he tries to be friends then I think I would see that as malicious and bullying.” (Male, study 3).

“Isolating a student from a friendship group – I believe there are various reasons for which a student can be isolated from a group – including by choice.” (Female, study 3).

Cyber-bullying was explored in detail in study two but less so in the other three studies. Most study two participants considered that cyber-bullying was just as harmful, or in some cases worse than, ‘traditional’ bullying due to the use of similar forms of “harassment,” “antagonizing,” “tormenting,” and ‘threatening’ through online platforms. Some young people believed that the physical distance between the victim and the bully is an important aspect of cyber-bullying:

“I think it’s worse because people find it easier to abuse someone when not face to face.” (Male, study 2).

“I think it could be worse, because lots of other people can get involved, whereas when it’s physical bullying it’s normally just between one or two or a smaller group, things could escalate too because especially Facebook, they’ve got potential to escalate.” (Female, study 2).

Other participants in study two spoke about bullying at school which transfers to an online platform highlighting no “escape” for some. In addition, it was made clearer that some young people considered distancing in relation to bullying and how this influences perceptions of severity:

“…when there’s an argument it can continue when you’re not at school or whatever and they can continue it over Facebook and everyone can see it then other people get involved.” (Female, study 2).

“I was cyber-bullied on Facebook, because someone put several hurtful comments in response to my status updates and profile pictures. This actually was extended into school by the bully…” (Male, study 2).

### Impact of Bullying on Victim

Although bullying behaviors were a primary consideration of young people’s understanding of bullying, many considered the consequences associated with bullying and in particular, the impact on mental health. In these examples, the specifics of the bullying event were irrelevant to young people and the focus was on how the behavior was received by the recipient.

In study two, young people divulged how cyber-bullying had adversely affected their ability to go to school and to socialize outside school. Indeed some young people reported the affects it had on their confidence and self-esteem:

“I developed anorexia nervosa. Although not the single cause of my illness, bullying greatly contributed to my low self-esteem which led to becoming ill.” (Female, study 2).

“It hurts people’s feelings and can even lead to committing suicide….” (Female, study 2).

Across the studies, young people who had been bullied themselves shared their individual experiences:

“….you feel insecure and it just builds up and builds up and then in the end you have no self-confidence.” (Female, study 2).

“…it was an everyday thing I just couldn’t take it and it was causing me a lot of anxiety.” (Male, study 4).

“I am different to everyone in my class …. I couldn’t take it no more I was upset all the time and it made me feel anxious and I wasn’t sleeping but spent all my time in bed being sad and unhappy.” (Male, study 4).

Young people who had not experienced bullying themselves agreed that the impact it had on a person was a large determiner of whether bullying had happened:

“When your self-confidence is severely affected and you become shy. Also when you start believing what the bullies are saying about you and start to doubt yourself.” (Female, study 3).

“…it makes the victim feel bad about themselves which mostly leads to depression and sadness.” (Male, study 2).

Further evidence around the impact of bullying was apparent in the data in terms of how relational aspects can affect perceived severity. In the case of cyber-bullying, young people suggested a sense of detachment because the bullying takes place online. Consequently, as the relational element is removed bullying becomes easier to execute:

“…because people don’t have to face them over a computer so it’s so much easier. It’s so much quicker as well cos on something like Facebook it’s not just you, you can get everyone on Facebook to help you bully that person.” (Female, study 2).

“Due to technology being cheaper, it is easier for young people to bully people in this way because they don’t believe they can be tracked.” (Male, study 2).

“The effects are the same and often the bullying can be worse as the perpetrator is unknown or can disguise their identity. Away from the eyes of teachers etc., more can be done without anyone knowing.” (Female, study 2).

Relational aspects of bullying were further highlighted with regards to how “banter” was understood, particularly with in-group bullying and how the same example can either be seen as “banter” or bullying depending on the nature of the relationship:

“…we’ve just been joking about, but it’s never been anything harsh it’s just been like having a joke. well, I haven’t done it but I’ve been in a crowd where people do it, so I don’t want to get involved just in case it started an argument.” (Female, study 3).

“But it also depends…who your groups with, for example, if I spoke to my friends from [School]… I wouldn’t like use taboo language with them because to them it may seem inappropriate and probably a bit shocked, but if I was with my friends outside of school we use taboo language, we’ll be ourselves and we’ll be comfortable with it, and if a stranger walked past and heard us obviously they’d be thinking that we’re being bullied ourselves.” (Female, study 3).

Furthermore, how individuals are perceived by others tended to influence whether they were believed or not. In study four for example, participants suggested that who the bullies were within the school might have impacted how complaints were acted upon by school officials:

“When I went to the school about it, the students said I had attacked them – all eight of them! I just realized that no one believes me….” (Female, study 4).

While in study three, a characteristic of bullying was the influence the aggressor has over the victim:

“When the victim starts to feel in danger or start to fear the other person. Consequently he or she tries to avoid the bad guy (or girl!)” (Male, study 3).

These relational and contextual issues also influenced a young person’s ability to report bullying.

### Reporting Bullying

Young people were more likely to report bullying when they considered it was ‘serious’ enough. Just under half of participants in study two sought emotional/practical support if they worried about, or were affected by cyber-bullying, with most talking to their parents. In study three, young people were less likely to seek support but when they did, most went to their teachers. In study four, all participants reported bullying in school where they did not feel supported.

Fear of making the bullying worse was captured across the studies as a reason for not reporting it:

“I’m scared that if I tell then the bullying will still go on and they will do more.” (Female, study 3).

“The bully might bully you if he finds out.” (Male, study 3).

Being able to deal with the incident themselves was also a reason for non-reporting:

“…it’s embarrassing and not necessary, my friends help me through it, adults never seem to understand.” (Female, study 2).

“I don’t tend to talk to anyone about it, I just keep it to myself and obviously that’s the worst thing you should ever do, you should never keep it to yourself, because I regret keeping it to myself to be honest….” (Female, study 3).

“…but I think I’d deal with it myself ‘cos. I was quite insecure but now I’m quite secure with myself, so I’ll sort it out myself. I think it’s just over time I’ve just sort of hardened to it.” (Male, study 3).

Most young people seeking support for bullying said they spoke to an adult but the helpfulness of this support varied. This finding is important for understanding relationships between young people and adults. Those who felt supported by their teachers for example, suggested that they took the time to listen and understood what they were telling them. They also reassured young people who in turn believed that the adult they confided in would know what to do:

“So I think the best teacher to talk to is [Miss A] and even though people are scared of her I would recommend it, because she’s a good listener and she can sense when you don’t want to talk about something, whereas the other teachers force it out of you.” (Female, study 3).

“My school has had assemblies about cyber-bullying and ways you can stop it or you can report it anonymously…. you can write your name or you can’t, it’s all up to YOU.” (Male, study 2).

Others however had a negative experience of reporting bullying and a number of reasons were provided as to why. Firstly, young people stated that adults did not believe them which made the bullying worse on some level:

“I went to the teachers a couple of times but, no, I don’t think they could do anything. I did sort of go three times and it still kept on going, so I just had to sort of deal with it and I sort of took it on the cheek….” (Male, study 3).

Secondly, young people suggested that adults did not always listen to their concerns, or in some cases did not take their concerns seriously enough:

“…I had had a really bad day with the girls so I came out and I explained all this to my head of year and how it was affecting me but instead of supporting me he put me straight into isolation.” (Male, study 4).

“I could understand them thinking I maybe got the wrong end of the stick with one incident but this was 18 months of me constantly reporting different incidents.” (Female, study 4).

“If cyber-bullying is brought to our school’s attention, usually, they expect printed proof of the situation and will take it into their own hand depending on its seriousness. However this is usually a couple of detentions. And it’s just not enough.” (Female, study 2).

Finally, some young people suggested that teachers did not always know what to do when bullying concerns were raised and consequently punished those making the complaint:

“I think I would have offered support instead of punishment to someone who was suffering with anxiety. I wouldn’t have seen anxiety as bad behavior I think that’s quite ignorant but they saw it as bad behavior.” (Male, study 4).

It is worth reiterating, that the majority of young people across the studies did not report bullying to *anybody*, which further underscores the contextual issues underpinning bullying and its role in enabling or disabling bullying behaviors. Some considered it was “pointless” reporting the bullying and others feared the situation would be made worse if they did:

“My school hide and say that bullying doesn’t go on cos they don’t wanna look bad for Ofsted.” (Male, study 2).

“My school is oblivious to anything that happens, many things against school rules happen beneath their eyes but they either refuse to acknowledge it or are just not paying attention so we must suffer.” (Female, study 2).

“That’s why I find that when you get bullied you’re scared of telling because either, in most cases the teacher will – oh yeah, yeah, don’t worry, we’ll sort it out and then they don’t tend to, and then they get bullied more for it.” (Female, study 3).

Young people were concerned that reporting bullying would have a negative impact on their friendship groups. Some were anxious about disrupting the *status quo* within:

“I think everyone would talk about me behind my back and say I was mean and everyone would hate me.” (Female, study 3).

Others expressed concern about the potential vulnerability they were likely to experience if they raised concerns of bullying:

“I was worried it might affect my other friendships.”(Boy, study 2).

“I’m scared that if I tell, then the bullying will still go on and they will do more.” (Female, study 3).

“….because they might tell off the bullies and then the bullies will like get back at you.” (Female, study 3).

These findings underscore the importance of contextual and relational factors in understanding bullying from the perspectives of young people and how these factors influence a young person’s ability or willingness to report bullying.

Finally one young person who had self-excluded from school due to severe bullying suggested that schools:

“…need to be looking out for their students’ mental wellbeing – not only be there to teach them but to support and mentor them. Keep them safe really… I missed out on about three years of socializing outside of school because I just couldn’t do it. I think it’s important that students are encouraged to stand up for each other.” (Female, study 4).

## Discussion

The studies presented in this paper illustrate the multitude of perceptions underpinning young people’s understandings of what constitutes bullying, both in terms of the behavior and also the impact that this behavior has on an individual. In turn, the ambiguity of what constitutes bullying had an impact on a young person’s ability to seek support. Discrepancies in bullying perceptions *within* and *between* young people’s groups are shown, highlighting the fluid and changing roles that occur within a bullying situation. Findings from quantitative studies have demonstrated the differing perceptions of bullying by adults and young people (see for example Smith et al., 2002; Vaillancourt et al., 2008; Maunder et al., 2010; Cuadrado-Gordillo, 2012). However, by combining findings from participatory research, new understandings of the relational and contextual factors important to young people come to the fore.

Young people participating in these four studies had unique knowledge and experiences of bullying and the social interactions of other young people in their schools and wider friendship groups. The underpinning participatory design enabled me to work alongside young people to analyze and understand their unique perspectives of bullying in more detail. The research teams were therefore able to construct meaning together, based not entirely on our own assumptions and ideologies, but including the viewpoint of the wider research participant group (Thomson and Gunter, 2008). Together, through the process of co-constructing bullying knowledge, we were able to build on what is already known in this field and contribute to the view that bullying is socially constructed through the experiences of young people and the groups they occupy (Schott and Sondergaard, 2014).

With regards to understanding what bullying is, the findings from these studies corroborate those of the wider literature from both paradigms of inquiry (for example Naylor et al., 2001; Canty et al., 2016); that being the discrepancies in definitions between adults and young people and also between young people themselves. Yet, findings here suggest that young people’s bullying definitions are contextually and relationally contingent. With the exception of physical bullying, young people did not differentiate between direct or indirect behaviors, instead they tended to agree that other contextual and relational factors played a role in deciding if particular behaviors were bullying (or not). The participatory research design enabled reflection and further investigation of the ideas that were particularly important to young people such as repetition and intentionality. Repetition was generally seen as being indicative of bullying being “serious,” and therefore more likely to be reported, and without repetition, a level of normality was perceived. This finding contradicts some work on bullying definitions, Cuadrado-Gordillo (2012) for example found that regardless of the role played by young people in a bullying episode (victim, aggressor or witness), the criteria of ‘repetition’ was not important in how they defined bullying.

Relational factors underpinning young people’s perception of bullying and indeed it’s “seriousness” were further reflected in their willingness or otherwise to report it. Fear of disrupting the *status quo* of the wider friendship group, potentially leading to their own exclusion from the group, was raised as a concern by young people. Some were concerned their friends would not support them if they reported bullying, while others feared further retaliation as a result. Friendship groups have been identified as a source of support for those who have experienced bullying and as a protective factor against further bullying (Allen, 2014). Although participants did not suggest their friendship groups are unsupportive it is possible that group dynamics underscore seeking (or not) support for bullying. Other literature has described such practices as evidence of a power imbalance (Olweus, 1995; Cuadrado-Gordillo, 2012) but young people in these studies did not describe these unequal relationships in this way and instead focused on the outcomes and impacts of bullying. Indeed Cuadrado-Gordillo (2012) also found that young people in their quantitative study did not consider “power imbalance” in their understanding of bullying and were more likely to consider intention. This paper, however, underscores the relational aspects of definitions of bullying and, how the dynamics of young people’s friendships can shift what is understood as bullying or not. Without such nuances, some behaviors may be overlooked as bullying, whereas other more obvious behaviors draw further attention. This paper also shows that contextual issues such as support structures can shift how young people see bullying. Contextual factors were evident across the four studies through the recognition of bullying being enabled or disabled by institutional factors, including a school’s ability to respond appropriately to bullying concerns. Young people suggested that schools could be influenced by bullies, perceiving them as non-threatening and consequently not dealing appropriately with the situation. Indeed some young people reported that their schools placed the onus on them as victims to change, consequently placing the “blame” on victims instead. These findings raise questions about who young people feel able to confide in about bullying as well as issues around training and teacher preparedness to deal with bullying in schools. Evidenced in these four studies, is that young people feel somewhat disconnected from adults when they have bullying concerns. Those who did report bullying, identified particular individuals they trusted and knew would support them. Novick and Isaacs (2010) identified teachers who young people felt comfortable in approaching to report bullying and described them as “most active, engaged and responsive.” (p. 291). The bullying literature suggests that as young people get older they are more likely to confide in friends than adults (Moore and Maclean, 2012; Allen, 2014). However, findings from this paper indicate that although fewer young people reported bullying, those who did confided in an adult. Young people have identified that a variety of supports are required to tackle bullying and that adults need to listen and work with them so nuanced bullying behaviors are not recognized as “normal” behaviors. Within the data presented in this paper, “banter” was portrayed as “normal” behavior. Young people did not specify what behaviors they regarded as “banter,” but suggested that when banter is repeated and intentional the lines are blurred about what is bullying and what is banter.

Exploring bullying nuances in this paper, was enhanced by the involvement of young people in the research process who had a unique “insider” perspective about what it is like to be a young person now and how bullying is currently affecting young people. In studies one and four, young people were “active respondents” (Bragg and Fielding, 2005) and provided adults with their own unique perspectives on bullying. It could be argued that study one did not involve the participation of young people. However, this study informed the basis of the subsequent studies due to the discrepancies noted in the literature about how bullying is understood between adults and young people, as well as the lack of young people’s voice and opportunity to participate in the reviewed research. Accordingly, young people’s data as “active respondents” informed adult understanding and led to future work involving more active research engagement from other young people. Participation in study four provided an opportunity for young people to contribute to future participatory research based on lived experiences as well as informing policy makers of the effects bullying has on the lives of young people (O’Brien, 2017). In studies two and three, young people were involved further along Bragg and Fielding (2005) continuum as “co-researchers” and “students as researchers” with these roles shifting and moving dependent on the context of the project at the time (O’Brien et al., 2018a). These young researchers brought unique knowledge to the projects (Bradbury-Jones et al., 2018) that could not be accessed elsewhere. Perspectives offered by the young researchers supported adults in understanding more about traditional and cyber-bullying from their perspectives. Furthermore, this knowledge can be added to other, quantitative studies to further understand why bullying happens alongside bullying prevalence, risk and protective factors, and negative outcomes.

## Conclusion

Findings from the four studies offer an alternative perspective to how bullying is understood by young people. Complexities in defining bullying have been further uncovered as understanding is informed by individual factors, as well as wider social and relational contexts (Horton, 2011; Schott and Sondergaard, 2014). This has implications for the type of support young people require. This paper highlights how definitions of bullying shift in response to relational and contextual aspects deemed important to young people. Because of this, further nuances were uncovered through the research process itself as the respective studies showed discrepancies in bullying perceptions *within* and *between* young people’s groups.

These understandings can act as a starting point for young people and adults to collaborate in research which seeks to understand bullying and the context to which it occurs. Furthermore, such collaborations enable adults to theorize and understand the complexities associated with bullying from the perspective of those at the center. There is a need for additional participatory research projects involving such collaborations where adults and young people can learn from each other as well as combining findings from different methodologies to enable a more comprehensive picture of the issues for young people to emerge. Further research is needed to unravel the complexities of bullying among and between young people, specifically in relation to the contextual and relational factors underscoring perceptions of bullying.

## Data Availability

The raw data supporting the conclusions of this manuscript will be made available by the authors, without undue reservation, to any qualified researcher.

## Ethics Statement

Ethical approval was granted for all four studies from the Faculty of Health, Education, Medicine and Social Care at the Anglia Ruskin University. The research was conducted on the premise of Gillick competency meaning that young people (in these studies over the age of 12 years) could consent for themselves to participate. Parents/carers were aware the study was happening and received information sheets explaining the process.

## Author Contributions

The author confirms being the sole contributor of this work and has approved it for publication.

## Conflict of Interest Statement

The author declares that the research was conducted in the absence of any commercial or financial relationships that could be construed as a potential conflict of interest.

## Footnotes

1 These findings focus on perceptions and data from the young people in the four studies. For a full discussion on adult perceptions please refer to the individual studies.
